# Is it possible to identify patient's sex when reading blinded illness narratives? An experimental study about gender bias

**DOI:** 10.1186/1475-9276-7-21

**Published:** 2008-08-18

**Authors:** Jenny Andersson, Pär Salander, Marie Brandstetter-Hiltunen, Emma Knutsson, Katarina Hamberg

**Affiliations:** 1 The Department of Public Health and Clinical Medicine, Family Medicine, Umeå University, 901 85 Umeå, Sweden; 2 Department of Social Work, Umeå University, 901 85, Umeå, Sweden; 3 Department of Radiation Science, Oncology, Umeå University, 901 85, Umeå, Sweden; 4 Department of Psychology, Umeå University, 901 85, Umeå, Sweden; 5 Centre for Gender Excellence at Umeå University, Research Programme Challenging Gender, 901 85, Umeå, Sweden

## Abstract

**Background:**

In many diseases men and women, for no apparent medical reason, are not offered the same investigations and treatment in health care. This may be due to staff's stereotypical preconceptions about men and women, i.e., gender bias. In the clinical situation it is difficult to know whether gender differences in management reflect physicians' gender bias or male and female patients' different needs or different ways of expressing their needs. To shed some light on these possibilities this study investigated to what extent it was possible to identify patients' sex when reading their blinded illness narratives, i.e., do male and female patients express themselves differently enough to be recognised as men and women without being categorised on beforehand?

**Methods:**

Eighty-one authentic letters about being diseased by cancer were blinded regarding sex and read by 130 students of medicine and psychology. For each letter the participants were asked to give the author's sex and to explain their choice. The success rates were analysed statistically. To illuminate the participants' reasoning the explanations of four letters were analysed qualitatively.

**Results:**

The patient's sex was correctly identified in 62% of the cases, with significantly higher rates in male narratives. There were no differences between male and female participants. In the qualitative analysis the choice of a male writer was explained by: a short letter; formal language; a focus on facts and a lack of emotions. In contrast the reasons for the choice of a woman were: a long letter; vivid language; mention of emotions and interpersonal relationships. Furthermore, the same expressions were interpreted differently depending on whether the participant believed the writer to be male or female.

**Conclusion:**

It was possible to detect gender differences in the blinded illness narratives. The students' explanations for their choice of sex agreed with common gender stereotypes implying that such stereotypes correspond, at least on a group level, to differences in male and female patients' illness descriptions. However, it was also obvious that preconceptions about gender obstructed and biased the interpretations, a finding with implications for the understanding of gender bias in clinical practice.

## Introduction

Gender plays an important, but not necessarily appropriate part, in medical decision-making. Research has shown that differences between men and women regarding biological processes, socioeconomic conditions, risk behaviour and environmental risk factors may all contribute to differences in health [1,2]. Thus it is sometimes appropriate to investigate and treat male and female patients differently. On the other hand, there is also evidence that women, for no apparent medical reasons, are not offered the same treatment as men, which raises the possibility of gender bias. For example, many studies show that women are less likely than men to receive more advanced diagnostic and therapeutic interventions [3-7]. In the clinical situation it is often difficult to know the extent to which gender differences in management reflect physicians' gender bias or are due to other physician, patient or communication characteristics related to gender [8-10].

Patients' wishes and communication behaviour contribute to gender differences in health care [11,12]. It is argued, for instance, that men describe their symptoms in a straightforward and demanding way while women often act in a submissive way during consultations, give vague descriptions of their symptoms and are hesitant to accept potentially dangerous measures such as surgery [7,13]. In studies of psychosocial adaptation to cancer a recurring result is that men, more than women, prefer to share information while women tend to adapt to the stressful situation by expressing and sharing emotions [14,15]. However, even if there is some tendency for men to be information-oriented and women emotion-oriented, the research results are more complex. There are reports showing that men experience distress when there are social constraints to express emotions [15], that the need for information is more pronounced among women [16], and that women are more knowledgeable about their disease than men [17].

### Perceptions about gender differences

Assumptions and beliefs about differences in men and women regarding behaviour, skills, emotions and needs are widespread in society. Assumed gender differences are often polarized as opposites, for example: males are associated with order, control and individualism while females are considered incapable of controlling their feelings and as having a natural sense for the family [18,19]. The man is seen as strong and active, and the woman as weak and passive; the man is symbolised by work, while the woman is associated with the home. In research, generalisations and stereotypes about men and women and other social groups are mainly treated as problematic since they bias interpretations of human activities and are sources of discrimination [20]. However, there is also an ongoing discussion about how much truth there might be in a given stereotype [21].

It is debatable whether there is a male and a female "language", but research shows that there are often gender differences in the way language is used [22,23]. Analyses of conversations show fairly clearly that men and women talk differently. Issues such as turn-taking, politeness, interruption, use of swear words etc. are distinctly gender marked and may be read as signifying power relationships [23-25]. Studies also provide evidence of differences in the way men and women write [26,27]. In her research on autobiographies, Mary Gergen found substantial differences in the narratives written by men and women. Men focused on the career and achievements of the subject [26]. Emotional ties were mentioned only as 'facts', i.e., male authors did not try to recreate in the reader emphatic emotional responses. On the other hand, in narratives written by women the career line was important but was mingled with other issues that had great personal impact. Furthermore, women's autobiographies dealt extensively with relationships with others.

Discourse processes have developed out of what and how things are told, who speaks (characterised for example by gender, age, ethnicity, education and social position), and who listens (characterized according to the same factors). Similarly, a gender perspective on written language includes not only the author and the narrative itself but also the reader's interpretation. Since readers of a text are usually aware of whether the author is a man or woman, their expectations and interpretations might be affected by their preconceptions about gender [26]. In an experiment involving an authentic text in two versions, identical in all but the simulated male or female author, differences were shown in how readers viewed the author. Based on the same text, the male writer was considered more trustworthy and intelligent and the female writer more humane [28]. This shows that preconceptions have a great influence on how a text is judged and gendered expectations of manstories and womanstories might be more important than content and facts [26].

In a previous study we investigated gender differences in 83 patients' letters concerning their experiences when being diagnosed with cancer [29]. It was found that more women than men wrote long, personal and emotional narratives, thus confirming the earlier results about gender differences in communication behaviour described above. However, the majority of letters, about 60%, were neither long, personal or emotional, nor short, impersonal or unemotional, and thus they were hard to categorize. When discussing these results we asked if the gender differences were significant enough to be detectable if all obvious hints about the patient's sex were removed (i.e., pronouns and expressions like "my wife" and "my husband")? If so, this would confirm that there are genuine differences in male and female patients' descriptions, differences that are not just creations of the reader knowing the sex of the patient-writer on beforehand.

The aim of the present paper was, therefore, to investigate the extent to which it was possible to identify the patients' sex by reading the same letters, once all information regarding the sex of the patient-writer had been removed. Students of psychology and medicine were invited to be participants. In order to provide nuances to the results, and hints about how gender was created, the students' explanations of their choice of sex were scrutinized in a few of the letters where the students had varying rates of success in determining the authors' sex.

## Method

### Letters from patients

The study was based on letters written by patients with a recent diagnosis of cancer. The letters were collected at an oncology department in Sweden during a five-month period in the late 90s, in order to analyse the manner in which the patients had received their diagnosis. All patients aged 18–70 who had received their cancer diagnosis 2–8 months previously, were asked to "...write a page or two describing how you received your diagnosis... including what the physician told you, how you reacted and how you felt afterwards. In addition, please describe both what you perceived as beneficial and what was detrimental..." Out of 187 consecutive patients invited, 138 (74%) submitted a written narrative [30].

In the present study, all the letters about breast cancer (n = 53) were excluded since this group of patients are subjected to a mammography-screening programme that was considered difficult to blind. Two letters were removed because they were illegible. The remaining letters (n = 83) were typed and all names, places and dates were systematically changed to prevent identification of patients, doctors and others concerned. To blind the letters all information that revealed the patient's sex was removed. The words "husband" and "wife" were consistently changed to "co-habiter", "mother" and "father" were changed to "parent". Detailed descriptions with references to specific clinics or surgical procedures were made less specific if they provided clues to the patient's sex. For example, any references to prostate or gynaecological symptoms or statements about women's clinics or urology departments were changed or removed. Abbreviations and spelling mistakes were retained as in the original letter. During this process two letters were excluded since it was considered too difficult to change them without distortion. Eighty-one letters remained, 42 written by men and 39 by women. The ethics committee of Umeå University approved the study.

### Participants

A total of 130 participants, 87 medical students and 43 psychology students at Umeå University, volunteered to take part in the study that was carried out on five occasions during the fall of 2005. The participants were aged 18 to 42 years (M = 24.4); 45 of them were men and 85 women.

In a pilot study we found that it was too demanding and time consuming for participants to read 81 letters. Therefore, two test-groups of participants where formed, groups A and B, each reading one half of the letters. The goal was to achieve an equal distribution of participants with respect to the number and sex in the two test groups (Table 1).

**Table 1:** Distribution of letters and characteristics of participants in the test groups.

	Letters (N = 81)			Participants (N = 130)		
	Number	Written by men/women		Number	Men/Women	Medicine/ Psychology
		
Group A	41	20/21		63	22/41	43/20
Group B	40	22/18		67	23/44	44/23

### Data collection

The participants were informed that the study was based on patients' authentic stories. Each participant was first asked to answer questions about their own sex, age and social background. They then read the letters belonging to their test group. For each letter they were asked to make a decision about the patient's sex (man or woman) and to explain their choice of sex in an open-ended question. The participants were instructed to read through the letters rapidly and make decisions based on their first impressions.

The current paper focuses on the decision about the author's sex and uses the explanations for the choice of sex in four letters to illuminate the complexity in the findings.

### Missing data

In total there were 5263 decisions about sex to be made, but data were missing for 79 of these decisions spread over letters and male and female participants. There is no reason to believe that this gap had any systematic influence on the results.

### Analysis

The relationships between participant characteristics (sex and discipline [medicine or psychology]) and participant success rate for the sex-decision task were analysed using the statistical program SPSS 11.0 for Windows. Unpaired t-tests and ANOVAs where used to compare differences between means. The level of significance was set at p < 0.05.

In order to shed more light on the statistical findings four letters were analyzed further. The letters that were selected were those with the highest and lowest frequencies of correct decisions about sex, together with two letters where about 50% of the participants' hade made a correct decision (see* in Table 3). For these letters the open-ended answers about the motives for the decision about sex were read and coded by three of the researchers (JA, MB-H, EK). In a joint session with all five researchers, the codes were compared, discussed and sorted into the broad categories: length, language and content. In a few cases of disagreement about how to categorize, the researchers discussed to find a solution. In this paper the motives for choosing a male or female patient are presented in typical examples, to illustrate the reasoning connected with each letter.

## Results

### Quantitative analysis

An independent t-test showed that there were no significant differences between the results of the medical and psychology students (p = 0.377) nor between the average numbers of correct decisions about sex in the test groups A and B (p = 0.250). There were also no significant differences between the success rates of male and female participants (p = 0.628).

Table 2 shows the percentage of correct decisions for all letters and male and female letters, made by all participants, and by male and female participants separately. The mean value for correct decisions among all participants for all letters was 61.7% with a variation from 26.8% to 82.5%. Comparing these results to chance, i.e. 50% correct decisions, independent t-tests showed that the participants were significantly better than chance in their judgements of male as well as female letters (p-values not shown in the table). The participants also chose 'male patient' significantly more often than 'female patient' (p < 0.000).

**Table 2:** Percentage of correct decisions about patient's sex made by male and female participants.

	All letters (N = 81)	Male letters (n = 42)	Female letters (n = 39)	p-values*
Male participants (n = 45)	61.2	65.7	55.9	< 0.000
Female participants (n = 85)	62.0	64.8	58.5	< 0.000
All participants (N = 130)	61.7	65.2	58.0	< 0.000

* p-values comparing the success for male and female letters.

Table 3 shows the distribution of letters according to the proportions of correct decisions made. For six of the 81 letters (7.4%) the proportion of correct decisions was lower than 30%, for 48 letters (59.3%) it was between 30 and 70 %, and for the remaining 27 letters (33.3%) the proportion of correct decisions was higher than 70%. Both male and female participants had a higher success rate on male than on female letters (p < 0.000).

**Table 3:** The letters sorted according to the percentage of correct decisions made about the patient's sex.

Correct decision interval (%)	Letter label	Number of letters (%)	Patient's sex^†^
0 – 5.0			
5.1 – 10.0	32*	1 (1.2)	2
10.1 – 15.0	59	1 (1.2)	2
15.1 – 20.0			
20.1 – 25.0			
25.1 – 30.0	4, 18, 25, 66	4 (4.9)	2, 2, 2, 2
30.1 – 35.0	44, 79, 36, 37,	4 (4.9)	1, 1, 1, 2
35.1 – 40.0	68	1 (1.2)	1
40.1 – 45.0	58, 55, 57	3 (3.7)	2, 1, 1
45.1 – 50.0	33, 81, 6, 23, 75, 2, 22, 74*	8 (9.9)	2, 2, 1, 1, 2, 1, 2, 1
50.1 – 55.0	45*, 50, 53, 54, 30, 39	6 (7.4)	2, 1, 2, 1, 2, 1
55.1 – 60.0	34, 13, 19	3 (3.7)	1, 2, 2
60.1 – 65.0	15, 29, 38, 48, 63, 5, 20, 46, 43, 62, 65, 73, 80	13 (16.0)	2, 2, 1, 2, 2, 2, 1, 2, 1, 1, 2, 2, 1
65.1 – 70.0	10, 14, 72, 56, 60, 69, 1, 11, 17, 35	10 (12.3)	2, 2, 1, 1, 1, 2, 1, 2, 1, 1
70.1 – 75.0	76, 7, 21, 52, 77, 31	6 (7.4)	1, 1, 1, 2, 1, 1
75.1 – 80.0	64, 78, 3, 9, 26, 41, 51	7 (8.6)	2, 1, 2, 1, 1, 2, 1
80.1 – 85.0	61, 71, 8, 40, 49	5 (6.2)	2, 2, 2, 1, 2
85.1 – 90.0	27, 12, 47, 67, 28, 42, 70	7 (8.6)	1, 1, 1, 1, 2, 1, 1
90.1 – 95.0	16	1 (1.2)	1
95.1 – 100	24*	1 (1.2)	2

Total		81 (100)	

^†^1 = man, 2 = woman

*Letters further examined in the qualitative analysis.

All six letters with a success rate below 30% were written by women. A majority of the letters (17/27) with a success rate above 70% were written by men.

### Qualitative examination

The students' explanations as to why they believed the author was a male or female patient varied from just a few words, e.g. "short letter" or "lot of emotions", to three or four sentences where they expressed several reasons and reflections. In the following, the letter on which the participants had the lowest proportion of correct decisions about sex is labelled 'the most difficult letter', the one with the highest proportion of correct answers is 'the easiest letter', and the two letters where the participants were quite divided on whether the author was a male or female patient are labelled the 'in-between letters'.

#### The most difficult letter

Letter 32, written by a woman, was very short and consisted of only two sentences:

"My cancer was detected in the following way: I had a severe cough and cold and blood started to come from the rectum."

Sixty participants read this letter. Five made a correct decision about sex and two of them explained their choice. They referred to "the sentence structure" and the disclosure of medical facts strongly linked to personal integrity as reasons for believing it was written by a woman patient.

Fifty-five participants thought that a man had written the letter and 46 of them explained their choice. Their reasons for a male author could be summarized as follows: The narrative was short and contained factual information and no emotions; the patient seemed dissociated from feelings and unwilling to share experiences and thoughts; several participants claimed that a female writer would have given a more balanced description and put some more effort into the assignment.

#### The easiest letter

The letter where the sex of the author was correctly identified most often (number 24), was written by a woman. It was longer than most letters, at 408 words, and was written in a format that described events in temporal order as well as the patient's reactions to these events. The patient mentioned family and friends, described medical staff and openly shared personal thoughts and feelings.

All 61 participants who read this letter correctly judged that the patient was a woman and 58 explained their choice. The length of the letter was often mentioned, the language was described as soft and vivid and the use of descriptions such as "smooth and gentle" about the male oncologist were frequently given as the motive for choosing a female writer. That the patient shared emotions, mentioned weakness and tears, described family members, friends and medical staff, and emphasized the meaning of support and network, were all seen as clues to the sex of the author. The attitudes of those around the patient were also seen as indicating a female author, e.g. when the patient was informed about the diagnosis, the doctor embraced and held the patient's hands.

#### The two 'in-between' letters

Letter 45 was written by a woman. The letter was 376 words long and started with the following sentence: *"You have asked for my experiences concerning the manner in which I received the information that I had malignant cell changes"*.

Following this the patient gave a detailed description of the course of events and the feelings involved. The letter also contained observations concerning positive and negative experiences of her treatment and how she was met by the staff.

Sixty-seven participants read this letter, and 35 of them (52%) correctly decided it was written by a woman. Sixty explained their choice. Participants who believed the author was a woman found the letter long and detailed. The language was described as proper, with an introduction ensuring that the reader did not forget why the letter was written. Some participants referred to the patient's use of the Swedish word "gräsligt", ("horrible" in English), as hinting that this was a female patient. The narrative was seen as emotional with disclosures of feelings of weakness and fear, seen as strong hints of a 'female writer'. Other reasons related to content concerned "the extensive description of the course of events" and the way the patient reflected on and analyzed the course of events.

Participants who thought that the writer was a man found the introduction formal. The sentences were described as short and the language academic. The use of the Swedish word "pallade" ("managed" in English) was associated with a man. The narrative was described as carefully prepared, based on factual information and focused on events rather than emotions. It gave the participants the impression of an evaluation, written on order. The patient seemed energetic and put more trust in himself than in those around him.

Letter 74 was written by a man. It consisted of 110 words and started with a description of the patient's shock when the black spot on the arm was diagnosed as a malignant tumour. The writer mentioned the doctor by title and full name and gave him credit for how the bad news was delivered. The patient described feelings of depression following the diagnosis. However, after receiving psychoactive drugs the patient felt a lot better and had returned to work.

Thirty-tree of the 67 participants (49%) who read the letter thought that the writer was a man while the other half thought it was a woman. Fifty participants explained their decision. Those who thought it was a man described the narrative as short and concise, distant and formal. They stated that the patient focused on the disease as a diagnosis rather than on the emotions involved. Other explanations focused on the patient's comment that it was important to get back to work, and the long waiting time before seeing a doctor.

The participants who believed it was written by a female patient thought, on the other hand, that a long waiting time to see a doctor indicated that the patient was a woman. They also found hints about gender in "the style" and "the choice of words". Other clues mentioned were how the patient referred to the doctor by title and full name and the comments about reception. But the most common motives for choosing 'female patient' were that the patient described anxiety and weakness and was not afraid to ask for help or medical treatment for depression.

## Discussion

### Summary

The results showed that in 62% of the cases the university student participants succeeded in identifying the sex of the patient who had written the letter, which was significantly more accurate than a chance allocation. Male and female students did not differ in this regard. The success rate varied between letters and for one third of the letters more than 70% of the participants made a correct decision. For the remaining two thirds of the letters many participants thus had problems identifying the patient's sex. There were significant differences between the students' success rate for male and female letters, with more correct decisions being made for male letters. In four letters the explanations for the choice of sex were analysed and the participants based their choices on three factors – length, language and content. The participants were more likely to say that a particular letter was written by a man if the letter was short, the language was more formal and academic, and the content focussed on the factual info. If the letter was long, written in more expressive manner, and described emotions and relationships the participants were more likely to decide that the author was a woman. However, depending on whether the participants believed the patient was a man or a woman, the same utterances and expressions were interpreted in different ways.

### On method

That the narratives were authentic and written by "real patients" with cancer, as opposed to constructed paper cases, increased the credibility of the study. Further, the letters were not initially collected with a gender study in mind but to study the communication of bad news. This fact, presumably, limited the risk that the patients consciously adjusted their narratives according to societal norms about male and female patients' behaviour.

In research comparing men and women there is always a risk of circular arguments. Men's and women's behaviours, thoughts or narratives are compared, and differences and similarities are noted. This raises the question of whether the interpretations are true differences or biased by the observers' preconceptions and expectations of a gendered pattern. A strength in this study was the use of the written narrative form, making it possible to create "neutral" patients in the sense that there was no obvious information within the text that revealed the patient's sex. On the other hand, differences between men and women in writing about illness might not be the same as differences when talking about illness, for example when seeing a physician. The interpretation of gender may be different in reading compared to listening. Thus the design with neutral patients and written narratives inherited weaknesses along with the strengths.

The students' participation in the study was voluntary and more women than men took part. However, comparisons of the results showed no significant differences in the responses of men and women students. The gender topic of the study may have contributed to a preponderance of male and female students with a special interest in gender issues. Whether this influenced the results is beyond our knowledge, but even participants aware of gender issues had to rely on their preconceptions and beliefs when sorting the letters.

The instructions to read through the letters rapidly and make judgements based on their first impressions forced the participants to be categorical. Many found this unpleasant, indicating an aversion towards using categorical generalisations. In studies of stereotypes and attitudes it is regularly found that people are inclined to express themselves in politically correct terms and try to distance themselves from gender stereotyping [31]. Yet, on an unconscious level they nevertheless rely on the stereotypes they are trying to avoid. Thus, if our students had felt less pressure it is likely that the same stereotypes would have emerged, but probably in more guarded terms.

### On results

#### Better than chance allocation

The participants made accurate decisions about the sex of the author in approximately 62% of the cases and in one third of the letters the success rate was even higher. These results indicate, hardly surprisingly, that there were gender differences in the illness narratives and confirmed our findings from a previous study that differences between male and female letters are detectable on a group level [29]. This finding is consistent with other studies that show fairly stable gender differences in conversations and use of language [23-26]. Reliable gender differences have also been found in meta-analyses of behaviours and traits in areas such as cognitive performance, cognitive attitudes, personality and group behaviour [21]. However, the fact that the students in our study were able to recognize gender differences, i.e. to make accurate decisions about the patient's sex in a majority of the blinded narratives, shows that knowledge and awareness of gender differences is common and widespread among people.

It is hard to know whether he success rate would have been different had the participants been qualified psychologists or physicians with more clinical experience. In a study with a similar design, English professors in fact did worse than college students when they were asked to identify whether a man or a woman had written different 100-word passages of American fiction [27]. Experience in reading or analysing texts did not increase their ability to categorize the author by sex.

In our study the success rate varied greatly across the letters showing that although preconceptions about gender differences rest on a basis of 'reality' on a group level, there are many exceptions and variations on the individual level. This fact illustrates the risk of making prejudiced assessments and biased interpretations in everyday communication.

The lack of a sex difference in students' ability to identify the patients' sex, i.e. to recognize gendered patterns in the narratives, is in line with previous research showing that on the whole men and women are aware of gender differences in behaviour to the same extent [21] and have similar associations and preconceptions about gender [31,32].

The participants were more successful at identifying which letters were written by male than female patients. One possible explanation for this might be related to 'the male norm', i.e., that men and behaviours associated with men are seen as the norm and the point of reference while women, and their needs and behaviour, are seen as exceptions in many situations [33]. One consequence of this might be that the participants were more inclined to see a man in the narratives and more prone to guess on a male writer when they were unsure. This would also explain why the participants chose 'male patient' more often. Another possible explanation is that women tend to show a greater capacity to vary their text than men, and to a greater extent adjust their language to existing circumstances [26]. In a social system where the man is the norm and women run the risk of being disregarded or not taken seriously, there might be a purpose behind women adjusting their language to a male language norm [24]. Some female writers may employ a discourse that seems like male writing, or at least seems to be in line with how readers are used to seeing male writing [27].

#### The decision process

When comparing the reasons for choosing a male or female writer in letters with the highest and lowest success rates, pros and cons of applying common generalizations about men and women in individual cases are illustrated. Letter 24 was written by a woman and fulfilled preconceptions about women's writing regarding length, personal and emotional content, and inclusion of family members and other people in the text. Subsequently, all participants succeeded in correctly identifying the author as a woman. Letter 32 was difficult since it was written by a woman whose letter did not fit into the stereotypes about women's way of communicating. On the contrary, she wrote 'like a man'. The participants commented that the author was 'writing briefly' and 'with factual information and no emotions', characteristics they associated with a male author. This indicates that the letters with very high rates of successful allocation corresponded with generalizations about gender while those with very low success rates clearly deviated from gender stereotypes.

The reasons underlying the allocation of the 'in-between' letters suggest that the participants were sifting and weighting a variety of factors when categorizing the author by sex. It appears that they created an image of the narrator's sex from some information they initially gleaned, and then they looked for clues to confirm their belief about the writer's sex. We do not know, however, to what extent these initial clues concerned 'length', 'language', 'content', or even something else, and we do not know the hierarchy governing these clues, i.e., was a 'long narrative' more likely to supersede the use of 'academic language' or 'emotional expressions' as cueing the initial impression that formed the basis for the decision. These are interesting questions that remain to be considered in forthcoming research. It was, nevertheless, striking that the same phenomenon or expressions were interpreted in quite different ways depending on whether the participant decided that the patient was a man or a woman. For example: the content in letter 45 was described as "emotional" or "reflective and analysing" by the participants who thought the author was a woman. The same content, on the other hand, was commented on as "based on facts and events" or "written on order like an evaluation" by the participants who thought the writer was a man. In the process, interpretations of identical utterances were thus biased by the participants' gendered preconceptions. These findings correspond with the results from earlier experiments, where identical articles gave the readers quite different views of the author depending on whether the simulated author was man or woman [28].

Our results were gathered in a study with an experimental design and the participants were students. It is reasonable to believe that similar interpretative processes take place in a clinical situation. In the clinic there are seldom doubts about a patient's sex and, depending on whether the patient is a man or a woman, the clinician has different preconceptions and expectations about the story that will be told. Thus, when a male patient describes his symptoms, needs and experiences, it is likely that the doctor, psychologist, or other health care staff member, will interpret and remember the narrative differently compared to when a female patient tells the same story [34,35]. This might be one clue to the mental processes causing the gender bias in diagnoses and treatment identified in many studies in various fields of health care [3-7,36,37].

The participants' explanations for their decisions contained many stereotypes, preconceptions and ideas about men and women. In the black-and-white generalizations that the participants uses the categories man and woman stood out as complete opposites defined by their difference. For example, participants explained their choice by comments such as "a woman wouldn't express herself this way". However, if stereotypical assumptions and generalizations were completely false and there were no differences between male and female writers, the participants would not have done significantly better than chance allocations in their judgements. Generalizations about men and women are based on individual experiences as well as abstractions and conceptions in society, and stereotypical ideas often contain some truth when compared to results from observational studies and other research [21]. In fact, people use generalizations and preconceptions as an aid in understanding the world. Without generalisations we would be lost in a fragmented social world that was difficult to understand. However, generalizations and stereotypes are also problematic as they bias what we see and hear; they imply a risk of neglecting variation, less well-known aspects, and of making skewed assessments.

To ensure that students in the health care field are aware of the risk of interpreting patients' behaviour in ways that reflect gender bias it seems essential to include reflections about the impact of preconceptions and generalisations in medical education. In healthcare, it is also vital to safeguard a working climate where generalisations and stereotypical attitudes towards men and women (and also other social groups, e.g. immigrants, unemployed, elderly and disabled) are constantly questioned and reflected on in clinical discussions among doctors, psychologists and other healthcare staff. The results from this article could contribute to such discussions, and the different interpretations of identical expressions depending on whether the author was taken for a man or a woman might be used as examples.

More knowledge about the cognitive, behavioural and communication processes leading to gender bias in medical work is needed. Observations of authentic consultations in different clinics would be valuable [38]. In a future study, we will focus on the explanations for the participants' choice of sex in more of the 'in-between' letters.

## Conclusion

It was possible for participants to detect gender differences in patients' illness narratives although the narratives were blinded. The reasons the participants gave for their sex-categorisation reflected common gender stereotypes suggesting that such stereotypes are more than problematic clichés; at least on a group level they correspond to differences in male and female patients' illness descriptions. However, it was also obvious that preconceptions about gender obstructed and biased the participants' interpretations of the letters. Depending on whether the participants believed the author was a male or a female patient they interpreted the same utterances in different ways. Translated into the clinical situation our results suggest that on the one hand there are gender differences that are recognizable and useful in clinical work consistent with common gender stereotypes. On the other hand, stereotyped preconceptions and generalisations about gender imply that there is a risk that health care staff might interpret a story told by a male patient differently than the identical story told by a woman, and this could result in gender biased investigations and treatments. These findings are important for further understanding of and research about gender bias processes in clinical work.

## Competing interests

The authors declare that they have no competing interests.

## Authors' contributions

All five authors contributed to the design, data collection and drafting of this article. JA, PS and KH were mainly responsible for the final drafting. All five authors read and approved the manuscript.
